# IL23R in the Swedish, Finnish, Hungarian and Italian populations: association with IBD and psoriasis, and linkage to celiac disease

**DOI:** 10.1186/1471-2350-10-8

**Published:** 2009-01-28

**Authors:** Elisabet Einarsdottir, Lotta LE Koskinen, Emma Dukes, Kati Kainu, Sari Suomela, Maarit Lappalainen, Fabiana Ziberna, Ilma R Korponay-Szabo, Kalle Kurppa, Katri Kaukinen, Róza Ádány, Zsuzsa Pocsai, György Széles, Martti Färkkilä, Ulla Turunen, Leena Halme, Paulina Paavola-Sakki, Tarcisio Not, Serena Vatta, Alessandro Ventura, Robert Löfberg, Leif Torkvist, Francesca Bresso, Jonas Halfvarson, Markku Mäki, Kimmo Kontula, Ulpu Saarialho-Kere, Juha Kere, Mauro D'Amato, Päivi Saavalainen

**Affiliations:** 1 Department of Medical Genetics, Biomedicum Helsinki, University of Helsinki, Helsinki, Finland; 2 Research Program for Molecular Medicine, Biomedicum Helsinki, Finland; 3 Department of Dermatology, Helsinki University Central Hospital and University of Helsinki, Helsinki, Finland; 4 Department of Medicine, University of Helsinki, Helsinki, Finland; 5 Department of Reproductive and Development Sciences, University of Trieste and IRCCS "Burlo Garofolo" Children Hospital, Trieste, Italy; 6 Heim Pal Children's Hospital, Budapest and University of Debrecen, Budapest, Hungary; 7 Paediatric Research Centre, University of Tampere Medical School and Tampere University Hospital, Tampere, Finland; 8 Department of Gastroenterology and Alimentary Tract Surgery, Tampere University Hospital and Medical School, University of Tampere, Tampere, Finland; 9 Faculty of Public Health, Department of Preventive Medicine, University of Debrecen, Debrecen, Hungary; 10 Faculty of Public Health, Department of Epidemiology and Biostatistics, University of Debrecen, Debrecen, Hungary; 11 Department of Surgery, Helsinki University Central Hospital, Helsinki, Finland; 12 Department of Gastroenterology, Helsinki University Hospital, Helsinki, Finland; 13 Department of Transplantation and Liver Surgery, University of Helsinki, Helsinki, Finland; 14 Department of Medicine, Karolinska Institutet, Stockholm, Sweden; 15 Department of Surgery, Karolinska Institutet, Stockholm, Sweden; 16 Department of Internal Medicine, Örebro University Hospital, Örebro, Sweden; 17 Karolinska Institutet, Department of Biosciences and Nutrition, Clinical Research Centre, Huddinge, Sweden

## Abstract

**Background:**

Association of the interleukin-23 receptor (*IL23R*) with inflammatory bowel disease (IBD) has been confirmed in several populations. *IL23R *also associates with psoriasis, suggesting that the gene may be an important candidate for many chronic inflammatory diseases.

**Methods:**

We studied association of single-nucleotide variants in *IL23R *with IBD in Swedish patients, in both Crohn's disease (CD) and ulcerative colitis (UC) subsets. The same genetic variants were also studied in Finnish patients with psoriasis or celiac disease, and in Hungarian and Italian patients with celiac disease.

**Results:**

Association of *IL23R *with IBD was replicated in our Swedish patients, and linkage and association of the *IL23R *region with psoriasis was found in the Finnish population. The *IL23R *region was also linked to celiac disease in Finnish families, but no association of *IL23R *variants with celiac disease was found in the Finnish, Hungarian or Italian samples.

**Conclusion:**

Our study is the first to demonstrate association of *IL23R *with CD and UC in Swedish patients with IBD. It is also the first study to report linkage and association of the *IL23R *region with psoriasis in the Finnish population. Importantly, this is the first report of linkage of the *IL23R *region to celiac disease, a chronic inflammatory condition in which *IL23R *has not been previously implicated.

## Background

Association of genetic markers in the interleukin-23 receptor (*IL23R*) gene, and the intergenic region between *IL23R *and *IL12RB2*, with inflammatory bowel disease (IBD) was first identified in 2006 [1]. This association has since been replicated in both Crohn's disease (CD) and ulcerative colitis (UC) [2-10], which are the two most frequent types of IBD, and also in paediatric CD [11-13], psoriasis [14-17] and ankylosing spondylitis [18]. However, no association of *IL23R *with CD could be found in the Japanese population [19]. *IL23R *does not appear to be associated with systemic lupus erythematosus [20], rheumatoid arthritis [21,22] or systemic sclerosis [23].

Our study set out to test the reported association of *IL23R *with IBD and psoriasis, using Swedish and Finnish patient materials. Given the chronic inflammatory nature of both conditions, as well as the tissue targeted by the immune response, we hypothesised that these same SNPs in the *IL23R *region might also play a role in celiac disease- a chronic inflammatory enteropathy caused by exposure to dietary gluten that can also manifest as a blistering cutaneous disease [24]. Association of *IL23R *with celiac disease was studied in three populations (Finnish, Italian and Hungarian) due to heterogeneous allele and/or haplotype frequencies in different population groups, where a functional variant may explain a significant part of disease risk in one population but not another.

## Methods

### Subject materials

#### Inflammatory bowel disease

Diagnosis of IBD (CD or UC) was assessed according to established clinical criteria, including endoscopic, radiology and histopathology data. The dataset consisted of 280 controls and 803 cases, of which 455 were UC cases and 348 were patients with CD. All patients were recruited at the Karolinska University Hospital in Stockholm; controls consisted of a random sample of ethnically-matched unselected individuals.

#### Celiac disease

The material consisted of 3 population datasets. From Finland, 260 families with celiac disease (185 multiplex families, 75 trios) and 165 single cases were collected at the University of Tampere. In most patients, celiac disease was diagnosed according to ESPGHAN criteria [25]. However, in a small number of cases, diagnosis was based only on the presence of disease-specific anti-endomysial antibodies (ca. 1% of cases). The 292 Finnish controls have been previously described [26]. The Hungarian dataset consisted of 400 families (204 multiplex families, 196 trios), 270 cases and 270 controls; they have been previously described [27]. The Italian dataset consisted of 139 cases and 198 controls. Untreated celiac patients were diagnosed in accordance with ESPGHAN criteria [25] and intestinal biopsies were analysed using Oberhuber's classification [28]. A subset of Finnish (10%) and Hungarian (12%) cases were also afflicted with dermatitis herpetiformis (DH), the blistering cutaneous manifestation of celiac disease [29]. Given that the genetic risk factors underlying the gut and skin pathologies of celiac disease are speculated to be distinct [27], linkage analyses were performed on the total patient cohort as well as stratifying the Finnish and Hungarian pedigrees by the presence or absence of DH. Information regarding DH was not available for Italian patients with celiac disease or for a minor subset of the Finnish families (27 families).

#### Psoriasis

The dataset consisted of 255 Finnish families (64 multiplex families, 191 trios), including 385 affected individuals, and has been described previously [30]. One psoriasis patient from each family was randomly chosen and analysed against the Finnish control material described previously [26].

Table 1 gives a summary of all sample datasets in this study. Written informed consent was obtained from all participants in this study and local ethics committees approved the study protocols.

**Table 1:** Sample sets in this study

**Disease**	**Sample set**		**Sample size**
*IBD*	Swedish case-controls	UC cases	455
		CD cases	348
		Controls	280

*Celiac disease*	Finnish families 260 families (185 multiplex, 75 trios)	Affected	607
		Unaffected	379
	
	Finnish case-controls	Cases	165
	
	Hungarian families 400 families (204 multiplex, 196 trios)	Affected	606
		Unaffected	474
	
	Hungarian case-controls	Cases	270
		Controls	270
	
	Italian case-controls	Cases	139
		Controls	198

*Psoriasis*	Finnish families 255 families (64 multiplex, 191 trios)	Affected	385
		Unaffected	516

*Controls*	Finnish controls (from Lappalainen *et al. *2008)	Controls	292

Datasets in this study. Multiplex families have more than one affected individual, trio families are comprised of father, mother, and an affected child. IBD indicates inflammatory bowel disease, UC indicates ulcerative colitis, and CD indicates Crohn's disease.

### Genotyping and analysis

Power calculations were performed using the online Genetic Power Calculator [31], with power estimated to replicate the results of Duerr *et al. *(2006) [1] in our TDT and case-control materials. The prevalence of IBD was set as 0.001, celiac disease set as 0.01 and psoriasis set as 0.02. The frequency of the rare protective allele was set as 0.07; the OR was set as 0.50 (and not 0.26 as in [1] because this risk effect is likely to be inflated).

SNP markers in the *IL23R *region were chosen from Duerr *et al. *(2006) [1] and were typed by MALDI-TOF analysis on the Massarray Analyzer platform (Sequenom, San Diego, USA). All genotyping was performed at the MAF core facility in Karolinska Institute, Stockholm, Sweden. Markers rs7517847 and rs1495965 were not typed due to technical difficulties. Genotyping results, pedigree structure and affection status were imported into a BC|GENE LIMS database (Biocomputing Platforms, Espoo, Finland).

Deviations from Mendelian inheritance in the family materials were determined with PEDCHECK 1.1 [32] and marker genotypes were discarded in families where Mendelian inheritance errors were detected. Mendelian inheritance error-rates for the pedigrees with celiac disease were 0.18% for the Finnish dataset and 0.15% for Hungarian; the error-rate for the Finnish pedigrees with psoriasis was 0.25%. Genotyping success rates and conformity to Hardy-Weinberg equilibrium (HWE) are shown in Additional file 1.

MERLIN 1.0.1 [33] was used to perform non-parametric linkage analysis in the Finnish and Hungarian pedigrees with celiac disease, as well as the Finnish pedigrees with psoriasis. The – pairs and – exp functions were used. Haploview 4.0 [34] was used to study linkage disequilibrium (LD) patterns, estimate haplotypes and perform association analysis. Odds ratios (OR) were calculated and two-sided p-values reported. Genehunter (version 2.1_r5 beta) [35] was used to perform transmission-disequilibrium tests (TDT) in the family materials. To extract information about association from the family materials without the presence of linkage, we utilised a script that randomly selected one affected individual from each family to be used as cases in a case-control type analysis (Biocomputing Platforms, Espoo, Finland).

The Cochran-Mantel-Haenszel (CMH) function in PLINK [36] was used to perform a meta-analysis of the three celiac disease datasets. The Breslow-Day test, estimating homogeneity of odds ratios, was non-significant (p > 0.05).

## Results

Additional file 2 shows the linkage disequilibrium (LD) patterns for the region surrounding *IL23R *and *IL12RB2 *in the HapMap dataset. The position of the single-nucleotide polymorphisms (SNPs) analysed in this study is also shown.

### *IL23R *and inflammatory bowel disease

The power of the IBD case-control dataset to replicate the findings of Duerr *et al. *(2006) was approximately 89%. The association of individual *IL23R *markers with IBD (UC, CD or both) is shown in Table 2A, with more detail in Additional file 3. The strongest associations were observed for SNPs rs11465804*G with the combined IBD dataset (CD and UC cases; p = 0.002, OR 0.42) and rs10489629*C with the UC cohort only (p = 0.002, OR 0.72).

**Table 2:** Single-marker association of *IL23R *to IBD, celiac disease and psoriasis

			rs1004819*T		rs10489629*C		rs2201841*C		rs11465804*G		rs11209026*A		rs1343151*C		rs10889677*A		rs11209032*A	
		*N (cases/controls)*	*p-value*	*OR*	*p-value*	*OR*	*p-value*	*OR*	*p-value*	*OR*	*p-value*	*OR*	*p-value*	*OR*	*p-value*	*OR*	*p-value*	*OR*

**A**	SWE IBD [UC&CD]	773/280	**0.004**	1.38	**0.006**	0.76	0.054		**0.002**	0.42	**0.003**	0.45	**0.014**	1.30	0.088		0.130	

	SWE IBD UC	461/280	**0.013**	1.36	**0.002**	0.72	**0.044**	1.27	**0.003**	0.42	**0.007**	0.43	**0.012**	1.34	0.059		0.111	

	SWE IBD CD	337/280	**0.006**	1.43	**0.049**	0.80	0.188		**0.022**	0.47	**0.038**	0.51	**0.039**	1.29	0.323		0.312	


**B**	HUN CEL	610/269	0.273		0.665		0.444		0.220		0.390		0.492		0.335		0.546	

	FIN CEL	460/292	0.400		0.773		0.401		0.874		0.503		0.247		0.499		0.634	

	ITA CEL	138/194	0.803		0.324		0.390		0.753		0.778		0.570		0.334		0.491	

	ALL CEL	1208/750	0.524		0.865		0.846		0.279		0.677		0.399		0.849		0.424	


**C**	FIN PSOR	250/292	0.853		0.203		0.619		0.186		0.357		0.776		0.808		0.582	

Association of SNPs in *IL23R *to inflammatory bowel disease (IBD), psoriasis (PSOR) and celiac disease (CEL) in cohorts from Sweden (SWE), Finland (FIN), Hungary (HUN) and Italy (ITA). Association to ulcerative colitis [UC] and Crohn's disease [CD] in the Swedish IBD (A); to celiac disease in the Finnish, Hungarian and Italian populations or combined material (B); and to psoriasis in the Finnish population (C). OR refers to odds ratios. Statistically significant p-values (p < 0.05) are highlighted in bold; p-values lower than 10^-3 ^are highlighted in bold and underlined. Detailed data are shown in Additional file 3.

Association of *IL23R *haplotypes with IBD is shown in Additional file 4. The haplotype TTCTGCAA was associated with risk of IBD in Swedish patients (p = 0.009, OR 1.38), while the CCTGATCG haplotype was associated with protection against IBD (p = 0.001, OR 0.41). It was not informative to calculate genotype association for rs11465804 or rs11209026 or the protective CCTGATCG haplotype because there were too few homozygotes for the rare protective allele.

### *IL23R *and celiac disease

The power of the TDT analysis to replicate the results of Duerr *et al. *(2006) [1] was approximately 64% in the Finnish and 82% in the Hungarian families with celiac disease. The power in the Finnish and Hungarian case-control datasets was estimated at 80% and 90%, respectively; it was 44% for the Italian case-control dataset.

The Finnish pedigrees with celiac disease demonstrated linkage to the *IL23R *region (max lod = 3.24, p = 0.00006, 135 informative families; Table 3A) as well as significant over-transmission of the rs10489629-rs201841-rs11465804-rs11209026 TTTG haplotype in presence of linkage (data not shown; 74 transmissions, 44 non-transmissions, p = 0.0005). When TDT without linkage was performed, this haplotype was not significantly over-transmitted to cases (40 transmissions, 31 non-transmissions; p = 0.28). The TTTG haplotype matches with the risk-associated alleles reported by Duerr *et al. *(2006) [1], with the exception of the T allele of rs2201841. The Hungarian pedigrees with celiac disease showed neither significant linkage to the SNPs in the *IL23R *region (lod = 0.4, p = 0.08, 132 informative families; Table 3A) nor significant association (data not shown).

**Table 3:** Linkage to *IL23R*

**A**	**Celiac disease**	**Samples**	**max Z-value**	**max lod**	**max lod p-value**
		Finnish families (135)	3.19	3.24	0.00006

		Hungarian families (132)	1.25	0.4	0.08

		Finnish and Hungarian families (268)	3.22	3.04	0.00009


**B**	**Celiac disease-DH vs. non-DH**	**Samples**	**max Z-value**	**max lod**	**max lod p-value**

		Finnish DH (34)	1.87	1.03	0.015

		Finnish non-DH (74)	2.53	1.71	0.0013

		Hungarian DH (11)	0.14	0.01	0.4

		Hungarian non-DH (121)	1.01	0.28	0.13


**C**	**Psoriasis**	**Samples**	**max Z-value**	**max lod**	**max lod p-value**

		Finnish families (51)	1.49	0.83	0.03

Linkage of *IL23R *to celiac disease and psoriasis. Max Z-value, max lod-value and max lod p-value are shown. The number of families informative for linkage is shown in brackets. Linkage to *IL23R *in A) Finnish and Hungarian celiac disease families as well as Finnish and Hungarian celiac disease families combined. B) Finnish and Hungarian celiac disease families with or without dermatitis herpetiformis (DH). C) Finnish psoriasis families.

The Finnish and Hungarian pedigrees with celiac disease were stratified on the basis of their co-presentation of dermatitis herpetiformis (i.e. families with DH or families without DH) and linkage was calculated. As shown in Table 3B, the Finnish families with DH (34 of whom were informative for linkage) yielded a non-parametric lodscore of 1.03 (p = 0.015), while the Finnish families without DH (74 were informative for linkage) yielded a non-parametric lodscore of 1.71 (p = 0.0013). The linkage signal (p = ns) in the Hungarian dataset came only from the families without DH (121 families informative for linkage). The Finnish pedigrees were also stratified into families with one or more individuals carrying the rare protective IBD allele (rs11209026*A) and families without this allele. Families with rs11209026*A comprised approximately 10% of the Finnish material and contributed to approximately 5% of the lodscore (p = ns, data not shown), suggesting that this rare allele does not explain the linkage in Finnish pedigrees with celiac disease.

One individual from each Finnish or Hungarian pedigree was added to the single case material to increase the power of the case-control analysis. The celiac disease case-control datasets showed no significant association to any of the markers at the *IL23R *locus, not as individual populations nor when combined for a meta-analysis (Table 2B and Additional file 3). The celiac disease material also demonstrated no association to the haplotypes of the SNPs in the *IL23R *region (Additional file 4).

### *IL23R *and psoriasis

The power of the Finnish psoriasis material to replicate the results of Duerr *et al. *(2006) [1] was > 63%. Only 51 of the 255 families with psoriasis were informative for linkage, yielding a lodscore of 0.83 (p = 0.03, Table 3C). However, the role of the *IL23R *locus in psoriasis was supported through the family-based association tests (TDT), in which all of the 255 families were informative. TDT without linkage showed significant under-transmission of the rs1343151-rs10889677-rs11209032 TCG haplotype to patients with psoriasis (14 vs. 35 transmissions, p = 0.002; data not shown); the corresponding rs11465804-rs11209026-rs1343151-rs10889677 TGCC haplotype was over-transmitted to patients (64 vs. 31 transmissions, p = 0.0007; data not shown). Importantly, the protective TCG haplotype fits with the results of Duerr *et al. *(2006) [1] and others; similarly, the risk TGCC haplotype also corresponds with previous studies, with the exception of the C allele of rs10889677.

An association analysis was performed by comparing cases with psoriasis (selected from the family material) with controls from [26]; it found no association of the *IL23R *locus with psoriasis (Table 2C, and Additional files 3 and 4). Neither the rs11465804-rs11209026-rs1343151-rs10889677 TGCC risk haplotype (0.164 frequency in cases and 0.165 in controls, p = 0.96) nor the rs1343151-rs10889677-rs11209032 TCG protective haplotype (0.537 frequency in cases, 0.549 in controls, p = 0.67) were associated with psoriasis in the Finnish case-control material.

## Discussion

Pathologies that are apparently unrelated have increasingly been shown to share common genetic risk factors, such as celiac disease and schizophrenia in terms of *MYO9B *[37,38]. Conditions that are driven by an overactive/inappropriate immune response can be more easily envisaged to share common factors that contribute to their chronic inflammatory state. It is for this reason that we have examined IBD, psoriasis and celiac disease in this study.

Our study is the first to show genetic association of *IL23R *with IBD in the Swedish population. In particular, we found strong association of the marker rs1004819 with Crohn's disease, thereby replicating the association reported in the German population [4]. In the German study, association was only demonstrated with this particular SNP, which is located at the junction of two linkage disequilibrium (LD) blocks (Additional file 2). These findings raise the possibility of another polymorphism in the LD block upstream of the rs1004819 marker that affects susceptibility to IBD. Such a polymorphism is envisaged to act independently of rs11209026 or the variants immediately surrounding it.

The risk and protective haplotypes associated with Crohn's disease and ulcerative colitis in the Swedish population were identical to those found in the first reported association of *IL23R *with IBD [1]. However, *IL23R *was not involved in susceptibility to ulcerative colitis in the Finnish population [26]. These findings are interesting given that the Swedish and Finnish are neighbouring populations and they also share association of Crohn's disease to the same genetic variants and risk/protective haplotypes at this locus. The differences in Swedish and Finnish cases with ulcerative colitis may reflect genetic variation in the two populations at other IBD risk loci.

The *IL23R *region demonstrated linkage to psoriasis in the Finnish pedigrees, suggesting that the locus is involved in this inflammatory skin disorder. Linkage of this gene region has not previously been shown to psoriasis in the Finnish population. Importantly, in support of our linkage results, association of *IL23R *with psoriasis was confirmed in the Finnish case-controls, demonstrating under-transmission of previously reported protective variants and over-transmission of the corresponding risk variants. Other studies have reported association to the rs11209026 (Arg381Gln) polymorphism in *IL23R*, as well as to polymorphisms in *IL12B*, which encodes the β-chain of the IL23 cytokine [14,15,39]. Taken together, these collective findings suggest that polymorphisms in several components of the IL23/IL23R-pathway may be important for susceptibility to psoriasis. Indeed, anti-IL12/IL23 antibody treatment ameliorates the symptoms of psoriasis [40], further supporting the importance of multiple components this pathway in the disease.

The gene region containing *IL23R *was strongly linked to celiac disease in the Finnish families, but did not reach statistical significance in the Hungarian pedigrees- a result that perhaps reflects the heterogeneity of the two populations. The discrepancy between the populations could raise questions about the biological relevance of the linkage findings, but large differences in allele and haplotype frequencies between populations have been reported by others, suggesting that the effect of the same genetic risk variant may vary considerably across different populations. Indeed, it is thought that some of the celiac disease risk loci for the Finnish and Hungarian populations are distinct [27]. Therefore, it is possible that the *IL23R *region is linked to celiac disease in the Finnish population but not the Hungarian. Future replication studies may help to determine whether the region is truly linked to celiac disease.

Interestingly, stratification of the celiac disease dataset by the co-occurrence of dermatitis herpetiformis indicated that linkage was independent of celiac disease sub-phenotype. Despite the linkage results in the Finnish families, the case-control material of all three populations (Finnish, Hungarian and Italian) showed no association of *IL23R *with celiac disease, and family-based association tests supported the lack of association of these markers.

The linkage of celiac disease to the *IL23R *region may be due to other genetic factors than the ones reported by Duerr *et al. *(2006) [1]. In support of this, stratification of the celiac disease dataset by the A-allele of rs11209026 indicated that linkage in the Finnish families with celiac disease was independent of this variant. Intriguingly, this allele A was recently found to be associated with risk instead of protection from both celiac disease and multiple sclerosis in the Spanish population [41]. These findings in the Spanish population suggest that, if rs11209026 is the primary functional variant, *IL23R *would play an opposite role in celiac disease and multiple sclerosis compared to IBD and psoriasis. Perhaps more likely, is that these opposite alleles are simply tagging distinct haplotypes carrying the real primary risk variants.

Variants in the LD block upstream of *IL23R *or in the downstream LD block covering *IL12RB2*, may explain the linkage to celiac disease in the Finnish population. *IL12RB2 *and *IL12RB1 *encode for the two chains of the IL12 receptor. The IL23 receptor shares the protein encoded by *IL12RB1 *but also requires *IL23R*. Similarly, the ligands bound by these receptors, IL12 and IL23, are composed by shared subunits: IL12 is encoded by *IL12A *and *IL12B*, while IL23 is encoded by *IL23A *and *IL12B*. Interestingly, celiac disease is associated with *IL12A *[42,43] and thereby differs from IBD and psoriasis, where *IL12B *variants have shown risk effect. Hence, the *IL12A *association and the proximity of *IL12RB2 *to *IL23R *(which appears linked to celiac disease), suggest that the genes of the IL12 pathway are particularly interesting to investigate in future genetic studies of celiac disease. Independent associations of psoriasis and psoriatic arthritis with both *IL23R *and a SNP 4 kb upstream of *IL12RB2 *[39], indicate that extended studies in this gene cluster would be relevant to other chronic inflammatory diseases as well.

*IL23R *gene variants have now been repeatedly implicated in a number of inflammatory diseases and perhaps suggest that these pathologies are mediated by the Th17 pathway. Despite many studies finding association of these diseases with the *IL23R *region, few have reported functional evidence for the involvement of *IL23R *variants. It is known that the rs11209026 SNP results in an amino acid change in the intracellular domain of the IL23 receptor and thus the polymorphism may affect the downstream signalling of the receptor. However, our preliminary functional studies suggest that the rs11209026 genotype does not significantly affect expression of the IL23 receptor on peripheral blood mononuclear cells of healthy donors (data not shown). Nevertheless, it cannot be excluded that other factors carried on the protective haplotype tagged by rs11209026 could have an effect on expression levels of IL23R, or on the stability or alternative splicing of mRNA.

It is apparent that the variants in the *IL23R *region examined in this study are not associated with celiac disease. However, it is equally clear that some factor in this gene region affects susceptibility to celiac disease. The region needs to be investigated further to identify the precise genetic variants that contribute to celiac disease. Future studies employing a tagging strategy of the whole *IL12RB2 *gene may be useful in IBD, psoriasis and celiac disease. Such a strategy may be able to elucidate the disease-causing variants in celiac disease, as well as identifying additional variants that modify the risk of IBD and psoriasis.

## Conclusion

Our study is the first to report association of *IL23R *with Crohn's disease and ulcerative colitis in Swedish patients with IBD. It is also the first study to demonstrate linkage and association of the *IL23R *region with psoriasis in the Finnish population. But perhaps the most interesting novel finding is that of linkage of the *IL23R *region to celiac disease. It remains to be elucidated whether *IL23R, IL12RB2 *or another gene at 1p31 confers risk to celiac disease.

## Competing interests

The authors declare that they have no competing interests.

## Authors' contributions

EE performed the genetic studies and wrote the manuscript. LK performed the genetic studies. ED performed the IL23R protein analysis. KKainu, SS, and US-K contributed to diagnosis and identification of psoriasis samples. ML performed genetic analysis on the Finnish IBD samples. FZ, TN, SV, and AV contributed to the diagnosis and identification of the Italian celiac disease samples. IK-S, RA, ZP, and GS contributed to diagnosis and identification of Hungarian celiac disease samples and controls. KKurppa, KKaukinen, and MM contributed to diagnosis and identification of Finnish celiac disease samples. MF, UT, LS, KKontula and PP-S contributed to diagnosis and identification of Finnish IBD samples. RL, LT, FB, JH, and MD'A contributed to diagnosis and identification of Swedish IBD disease samples. MM, KKontula, US-K, JK and PS participated in the design and coordination of the study, and PS, MD'A and KKontula funded it. PS and MD'A conceived of the study and participated in writing the manuscript. All authors read and approved of the final manuscript.

## Pre-publication history

The pre-publication history for this paper can be accessed here:



## Supplementary Material

Additional file 1 **Genotyping call rates and HWE.** Genotyping success rate and quality of genotypes A) Genotyping call rates for each dataset and *IL23R *marker. All markers in the Swedish IBD dataset showed a call rate of over 95%, all markers in the Finnish psoriasis dataset had a call rate over 96%, and all markers in the celiac materials had a call rate of over 92%. B) Conformity to the Hardy-Weinberg equilibrium (HWE) in our datasets for each *IL23R *marker. Some of the markers showed borderline significant deviation (p = 0.01–0.05) from HWE (underlined), but no marker had a HWE p-value less than 0.05 in more than one dataset, so all markers were used for the association analysis. Click here for file

Additional file 2 **LD patterns of *IL23R*. LD patterns of the region around the *IL23R *gene in the CEU population (from Hapmap build 36 ).** 67400 K to 67640 K of chromosome 1 is shown. Dark colour indicates high LD; lighter colour indicates less LD. The position of the eight SNP markers analysed in this study is shown. Click here for file

Additional file 3 **Supplementary single-marker association results.** Association to single markers in the *IL23R *region. The table shows the sample size of each dataset, the genotype counts and frequency of both SNP alleles in cases and controls in each marker, as well as p-values, odds ratios (OR) and the 95% confidence intervals for the odds-ratios (CI). Association to inflammatory bowel disease (IBD), psoriasis (PSOR) and celiac disease (CEL) in cohorts from Sweden (SWE), Finland (FIN), Hungary (HUN) and Italy (ITA). Statistically significant p-values (p < 0.05) are highlighted in bold; p-values lower than 10^-3 ^are highlighted in bold and underlined. Click here for file

Additional file 4 **Haplotype association of *IL23R *to IBD and celiac disease.** Association of *IL23R *haplotypes to inflammatory bowel disease (IBD), psoriasis (PSOR) and celiac disease (CEL) in cohorts from Sweden (SWE), Finland (FIN), Hungary (HUN) and Italy (ITA). Association to ulcerative colitis [UC] and Crohn's disease [CD] in the Swedish IBD material (A); to celiac disease in the Finnish, Hungarian and Italian populations (B); and to psoriasis in the Finnish population (C). OR refers to odds ratios, 95% CI to the OR 95% confidence interval. Statistically significant p-values (p < 0.05) are highlighted in bold. Click here for file
